# Supplementary material for: Effects of Multiple Environmental Stressors on Zoobenthos Communities in Shallow Lakes: Evidence from a Mesocosm Experiment
Source: Animals (Basel). 2023 Dec 1;13(23):3722. doi: 10.3390/ani13233722 (PMC10705673; doi:10.3390/ani13233722)
Supplement: Supplementary file 1 [file animals-13-03722-s001.zip › Table S1.pdf]

Table S1. Effects of temperature, eutrophication, pesticide, and their interactions on zoobenthos abundance at the end of the experiment. Accumfreq represents the cumulative proportion of species abundance. "***" represents highly significant ($p \leq 0.001$), "**" represents significant ($0.001 < p \leq 0.01$), "*" represents moderately significant ($0.01 < p \leq 0.05$), "." represents approaching signif-icance ($0.05 < p \leq 0.1$), "NS" represents not significant ($p > 0.1$); "(+)" represents positive correlation ($r > 0$), "(-)" represents negative correlation ($r \leq 0$).

Species name	Rank	Abundance	Accumfreq	Warming	Eutrophic	Pesticide
Chironomus sp.	1	1820	15.5	** (+)	NS	*** (+)
Glyptotendipes sp.	2	1690	29.9	NS	NS	* (-)
Ecnomus sp.	3	1497	42.6	NS	NS	*** (-)
Radix swinhoei	4	1063	51.7	*** (+)	* (+)	*** (+)
Limnodrilus hoffmeisteri	5	881	59.2	* (-)	** (+)	** (-)
Glossiphonia lata	6	600	64.3	NS	NS	* (+)
Hippeutis umbilicalis	7	463	68.2	NS	NS	* (+)
Polypedilum sp.	8	452	72.1	NS	NS	NS
Limnodrilus sp.	9	391	75.4	NS	NS	NS
Bellamya sp.	10	334	78.2	NS	NS	NS
Branchiura sowerbyi	11	322	81	NS	NS	*** (-)
Sympetrum sp.	12	251	83.1	. (+)	NS	NS
Limnodrilus claparedianus	13	247	85.2	NS	NS	NS
Alocinma longicornis	14	215	87	NS	NS	NS
Parachironomus sp.	15	211	88.8	NS	NS	*** (+)
Bellamya_aeruginosa	16	167	90.3	NS	NS	NS
Ischnura sp.	17	143	91.5	NS	NS	NS
Parafossarulus striatulus	18	131	92.6	NS	NS	NS
Cricotopus trifasciatur	19	108	93.5	NS	NS	*** (+)
Pila sp.	20	104	94.4	NS	NS	* (+)
Assiminea sp.	21	103	95.3	NS	NS	NS
Bithynia fuchsiana	22	91	96	NS	NS	. (+)
Bellamya angularia	23	64	96.6	. (-)	NS	NS
Ablabesmyia sp.	24	53	97	NS	NS	NS
Bellamya purificata	25	50	97.5	NS	NS	NS
Paludinella stricta	26	39	97.8	NS	NS	. (+)
Orthocladius sp.	27	20	98	NS	NS	* (+)
Hippeutis cantori	28	17	98.1	NS	NS	NS
Bithynia misella	29	16	98.2	NS	NS	NS
Radix plicatula	30	15	98.4	NS	NS	NS
Physa acuta	31	14	98.5	NS	NS	NS
Tanytarsus sp.	32	14	98.6	NS	. (+)	NS
Parapoynx sp.	33	14	98.7	. (+)	NS	. (+)
Limnodrilus grandisetosus	34	12	98.8	NS	NS	NS
Cloeon sp.	35	11	98.9	NS	NS	* (-)
Paracercion sp.	36	11	99	NS	NS	NS
Chironomidae pupa	37	11	99.1	NS	NS	NS
Cricotopus sp.	38	10	99.2	NS	NS	NS
Barbronia weberi	39	8	99.3	NS	NS	NS
Polypylis hemisphaerula	40	7	99.3	NS	NS	NS
Paraplea sp.	41	6	99.4	NS	NS	NS

Bellaamya dispiralis	42	5	99.4	NS	NS	NS
Clinotanypus sp.	43	5	99.5	NS	NS	NS
Gyraulus albus	44	5	99.5	NS	NS	NS
Nigrobaetis sp.	45	5	99.5	NS	NS	NS
Tanypus sp.	46	5	99.6	NS	NS	NS
Harnischia sp.	47	5	99.6	NS	NS	NS
Rheopelopia sp.	48	5	99.7	NS	NS	NS
Bellamya quadrata	49	4	99.7	NS	NS	NS
Ephydridae	50	4	99.7	NS	NS	NS
Pseudothemis zonata	51	3	99.8	NS	NS	NS
Macrobrachium sp.	52	3	99.8	NS	NS	NS
Dicrotendipes sp.	53	3	99.8	NS	NS	NS
Parafossarulus eximius	54	3	99.8	NS	NS	NS
Orthetrum sp.	55	3	99.9	NS	NS	. (-)
Anax parthenope	56	2	99.9	NS	NS	NS
Procladius sp.	57	2	99.9	NS	NS	NS
Caenis sp.	58	1	99.9	NS	NS	NS
Glossiphonia complanata	59	1	99.9	NS	NS	NS
Anodonta angula	60	1	99.9	NS	NS	NS
Limnodrilus udekemianus	61	1	99.9	NS	NS	NS
Valvata sp.	62	1	99.9	NS	NS	NS
Baetis sp.	63	1	100	NS	NS	NS
Nais inflata	64	1	100	NS	NS	NS
Polypedilum scalaenum	65	1	100	NS	NS	NS
Hydrophilus sp.	66	1	100	NS	NS	NS
Paratanytarsus sp.	67	1	100	NS	NS	NS
Hydrobaenus sp.	68	1	100	NS	NS	NS
